# The Use of Bivariate Spatial Modeling of Questionnaire and Parasitology Data to Predict the Distribution of *Schistosoma haematobium* in Coastal Kenya

**DOI:** 10.1371/journal.pntd.0002016

**Published:** 2013-01-24

**Authors:** Hugh J. W. Sturrock, Rachel L. Pullan, Jimmy H. Kihara, Charles Mwandawiro, Simon J. Brooker

**Affiliations:** 1 Faculty of Infectious and Tropical Diseases, London School of Hygiene and Tropical Medicine, London, United Kingdom; 2 Eastern and Southern Africa Centre of International Parasite Control, Kenya Medical Research Institute (KEMRI), Nairobi, Kenya; 3 KEMRI-Wellcome Trust Research Programme, Nairobi, Kenya; National Institute of Parasitic Diseases, Chinese Center for Disease Control and Prevention, China

## Abstract

**Background:**

Questionnaires of reported blood in urine (BIU) distributed through the existing school system provide a rapid and reliable method to classify schools according to the prevalence of *Schistosoma haematobium*, thereby helping in the targeting of schistosomiasis control. However, not all schools return questionnaires and it is unclear whether treatment is warranted in such schools. This study investigates the use of bivariate spatial modelling of available and multiple data sources to predict the prevalence of *S. haematobium* at every school along the Kenyan coast.

**Methodology:**

Data from a questionnaire survey conducted by the Kenya Ministry of Education in Coast Province in 2009 were combined with available parasitological and environmental data in a Bayesian bivariate spatial model. This modeled the relationship between BIU data and environmental covariates, as well as the relationship between BIU and *S. haematobium* infection prevalence, to predict *S. haematobium* infection prevalence at all schools in the study region. Validation procedures were implemented to assess the predictive accuracy of endemicity classification.

**Principal Findings:**

The prevalence of BIU was negatively correlated with distance to nearest river and there was considerable residual spatial correlation at small (∼15 km) spatial scales. There was a predictable relationship between the prevalence of reported BIU and *S. haematobium* infection. The final model exhibited excellent sensitivity (0.94) but moderate specificity (0.69) in identifying low (<10%) prevalence schools, and had poor performance in differentiating between moderate and high prevalence schools (sensitivity 0.5, specificity 1).

**Conclusions:**

Schistosomiasis is highly focal and there is a need to target treatment on a school-by-school basis. The use of bivariate spatial modelling can supplement questionnaire data to identify schools requiring mass treatment, but is unable to distinguish between moderate and high prevalence schools.

## Author Summary

The highly focal nature of schistosomiasis means that treatment is most cost-effective when delivered on a school-by-school basis. Questionnaires of reported blood in urine (BIU) distributed through the existing school system are a rapid, valid method to classify schools according to WHO treatment thresholds. Their usefulness, however, can be hampered by incomplete return rates that impede treatment decisions in schools with missing questionnaire data. Using data from coastal Kenya, this study describes the use of Bayesian spatial modeling that combines questionnaire data with available parasitological data to make predictions of *S. haematobium* infection prevalence at all schools. Results showed that reported BIU was highly focal and was negatively associated with the distance to the nearest river. The final model was able to discriminate between schools that require treatment or not, but was less reliable at distinguishing between medium and high prevalence schools. Similar Bayesian spatial models may prove useful for modeling and predicting other diseases where multiple diagnostic techniques are employed.

## Introduction

The pharmaceutical industry and the global health community have recently committed to providing up to 250 million tablets of praziquantel each year for treatment of schistosomiasis [1]. It is essential that these treatments are targeted to schools and communities at greatest need, based on up-to-date epidemiological information. Whilst challenges still exist in the rapid assessment of *Schistosoma mansoni* prevalence [2], [3], [4], the use of morbidity questionnaires on reported blood in urine (BIU) to identify schools/communities with a high prevalence of *S. haematobium* is a well-established and reliable approach, based on extensive validation [3], [4], [5]. Such questionnaires can readily be distributed through the existing school system at sub-national and national levels [6], [7], [8]. However, a challenge often encountered when relying upon questionnaires distributed via the education system is incomplete return of questionnaires by schools from local to central levels [4]. Consequently, there is little basis upon which to base treatment decisions for those schools with missing questionnaire data.

One possible solution to this problem is to predict the prevalence of *S. haematobium* on the basis of either questionnaire data from neighbouring schools or environmental factors known to influence infection risk [9], or a combination of both factors. The spatial modeling of schistosomiasis is predicated on the sensitivity of snail intermediate hosts to variations in temperature and rainfall [9], [10], [11] and such environmental factors, along with proxy variables such as vegetation index, have successfully been used to explain and predict *S. haematobium* risk in a number of settings [9], [12], [13], [14]. For example, Clements *et al.* [7] used Bayesian geostatistics to model available area-level data on the percentage of school children reporting blood in urine from Tanzania with information on urban-rural status, elevation and vegetation index, to predict infection prevalence in those administrative unit (wards) with insufficient data. This study did not, however, include any validation of the reliability of the blood in urine data, using parasitological data. In mapping *Loa loa* in west Africa, Crainiceanu *et al.* [15] developed a bivariate spatial model to calibrate the RAPLOA methodology and to map the spatial variation in the predictive probability that prevalence of *L. loa* infection exceeds a predetermined policy intervention threshold. Here we adapt this modelling approach to predict the prevalence of *S. haematobium* at schools without infection data throughout coastal Kenya, using both BIU data available for schools which returned questionnaire results to the Kenya national deworming programme and parasitological data from a validation study [8]. Modeling BIU and parasitological data together makes it possible to predict *S. haematobium* prevalence at schools for which this information is missing, which would not be possible if modeling questionnaire data alone.

## Methods

### Data

**Figure 1:**
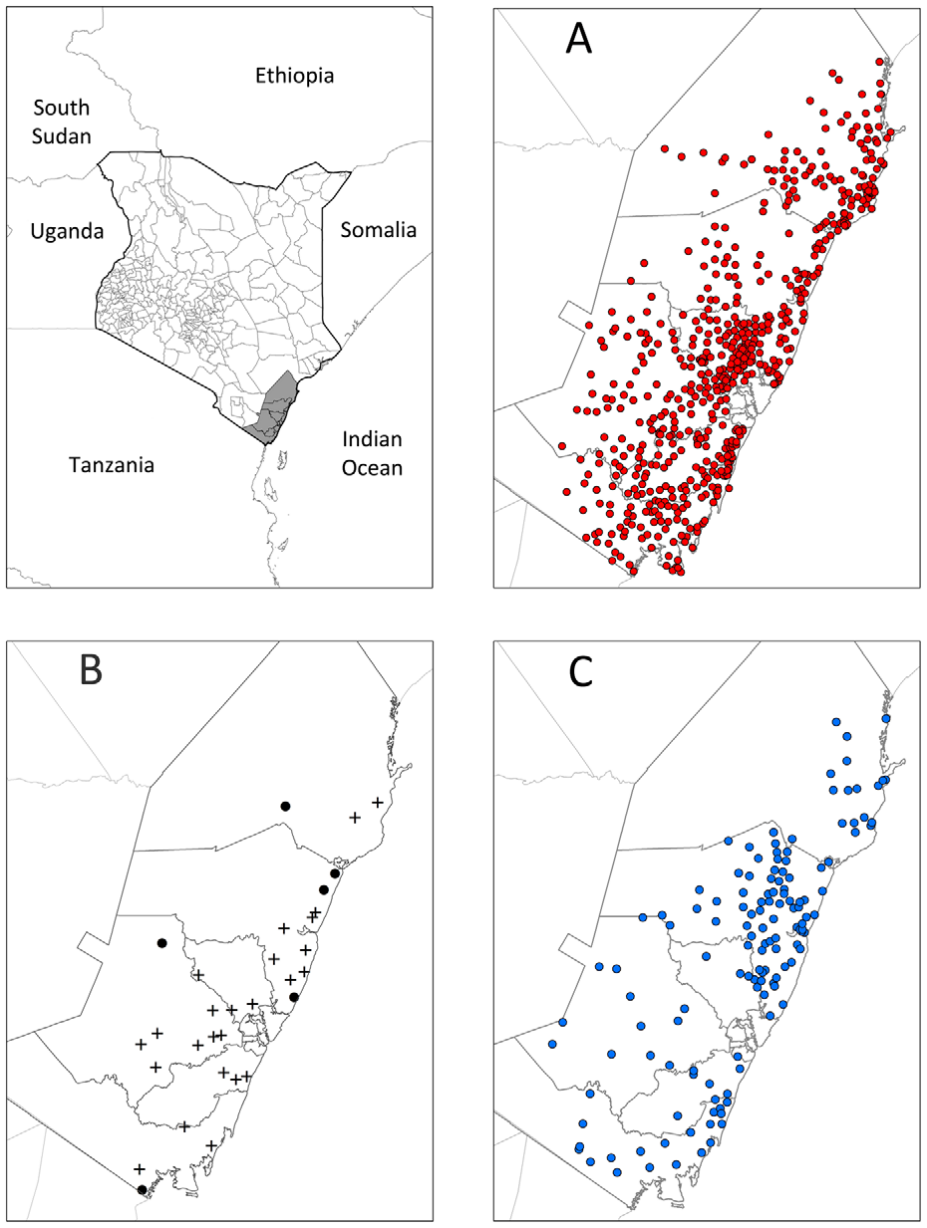
The location of the study districts (inset map) and schools (main map) in Kenya. (A) Locations of schools with questionnaire data, (B) Locations of schools with parasitological data (crosses show schools also with questionnaire data and circles represent schools with parasitological data only), and (C) Locations of schools with no data.

**Figure 2:**
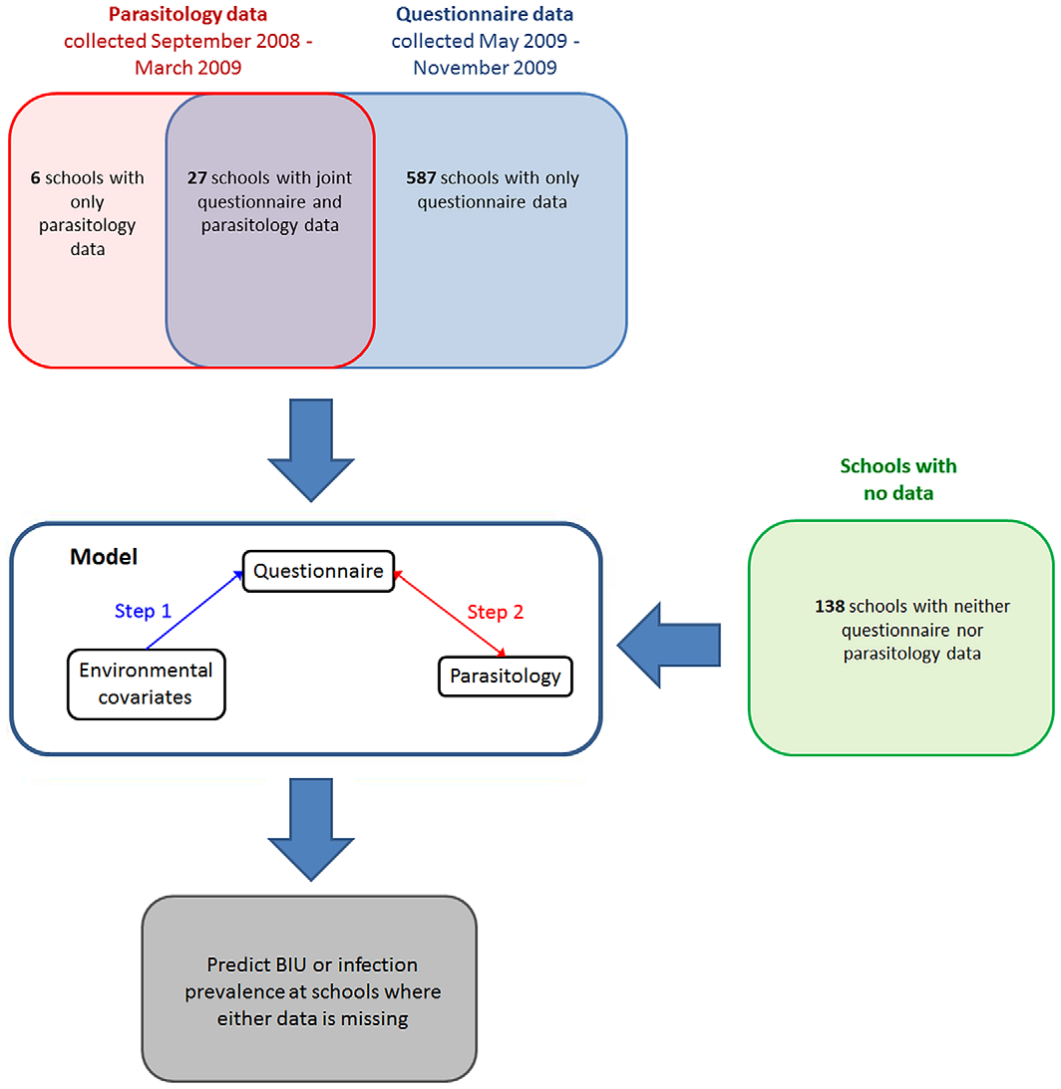
Schematic of the Bayesian model. The model is comprised of three components reflecting the different possible combinations of available data at each location: i) schools for which only questionnaire and environmental data are available and used to inform the parameters governing the relationship between the two (step 1); ii) schools which have both questionnaire and parasitology are used to inform the relationship between the proportion of children reporting blood in urine and the prevalence of *S. haematobium* (step 2); iii) schools at which only parasitology and covariate data are available are used to further inform the model by identifying parameters values of steps 1 and 2 that best explain the link between environmental covariates and infection prevalence. Within the Bayesian framework, these three components are solved simultaneously, making it possible to find the parameter values for steps 1 and 2 that best fit all available data.

Two distinct datasets relating to urinary schistosomiasis in schools across six districts in Coast Province, Kenya were available for analysis (Figure 1): i) parasitological data for 33 schools on the prevalence of *S. haematobium*, based on urine filtration; and ii) data on the proportion of school children reporting BIU for 613 schools, based on a survey distributed by the Ministry of Education to all schools. Details of these datasets and their collection are outlined elsewhere [8]. Briefly, parasitological surveys were conducted between September 2008 and March 2009. In a random selection of schools, selected with probability proportional to the population of the district, 10 boys and 10 girls were selected from each of classes 2–6 to supply a urine sample, which was filtrated through polycarbonate membrane filter (10 mls) and examined for presence of *S. haematobium* eggs. Between May 2009 and November 2009, the Ministry of Education distributed BIU questionnaires, which were administered by teachers in schools and collated by head teachers for forwarding to the district education office which then sent collated district-level results to Nairobi for analysis. Of the 33 schools with parasitology data, 27 also returned questionnaire data. In total, therefore, there were 587 schools with only questionnaire data, 27 schools with joint questionnaire and parasitology data, 6 schools with only parasitology data, and 138 without any data (Figure 1, Figure 2), making a total of 758 schools.

School-level parasitological and questionnaire data were related to a variety of high-resolution environmental data. Maximum land surface temperature, altitude and precipitation at 30-arcsec (∼1 km) resolution were taken from the WorldClim website [16]. Distance to nearest river was estimated in ArcMap 10 from an electronic map obtained from HydroSHEDS project [17]. Enhanced vegetation index (EVI; a measure of vegetation density) for 2001–2005 were obtained from the Moderate Resolution Imaging Spectroradiometer (MODIS) [18] and global land-cover type was estimated using electronic maps generated by the GlobCover at 300 m resolution for the year 2005 [19]. Scatter plots revealed a non-linear relationship between infection and distance to nearest river and based on observed data three categories of distance were generated, <2, 2–5 and ≥5 km. A likelihood ratio test revealed that distance to water categorized in this way provided a better model fit than when included as a linear (p = 0.038) or quadratic term (p<0.001).

### Statistical analyses

The aim of the analysis was to predict the prevalence of *S. haematobium* infection in schools for which there were no parasitology data, using all available questionnaire and parasitology data. To do this, we used Bayesian model-based geostatistics (MBG) to model the proportion of children reporting blood in urine on the basis of environmental covariates, whilst simultaneously modeling the relationship between the questionnaire data and parasitological data from schools where both sets of information were available (Figure 2).

**Figure 3:**
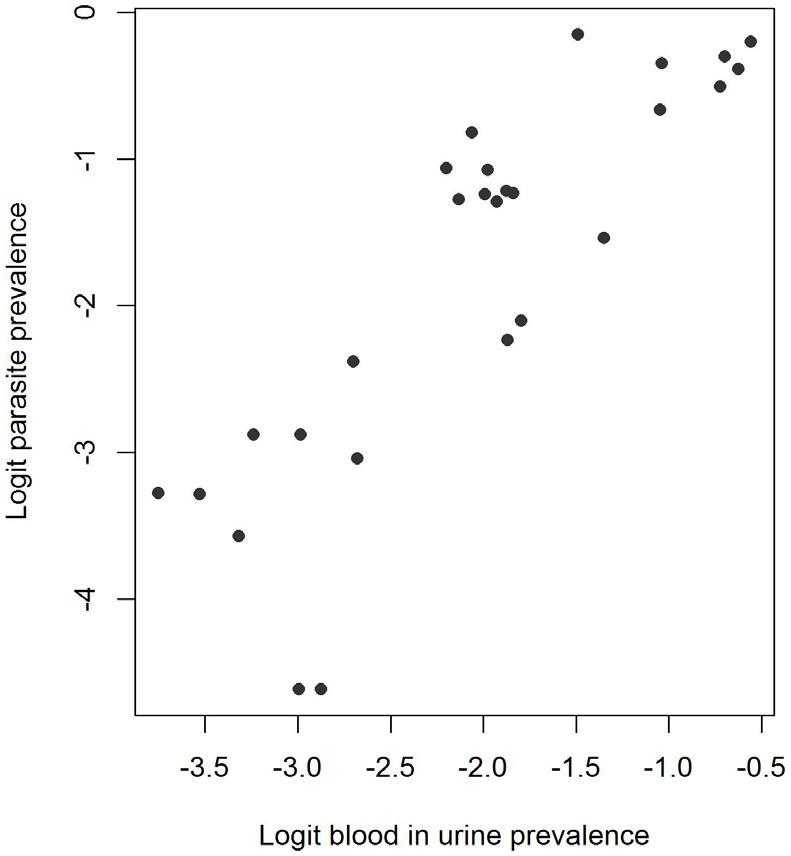
Relationship between the prevalence of reported blood in urine and prevalence of *S. haematobium*. Data are displayed following an empirical logit transformation.

A bivariate binomial model for the number of children reporting BIU and infected with *S. haematobium* was fitted as follows:
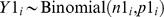


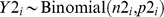


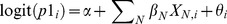
(1)

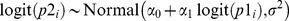
(2)where 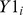 and 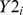 are the numbers positive according to questionnaire and parasitology respectively, 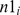 and 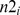 the number of individuals sampled using questionnaire and parasitology and 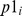 and 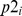 the proportion positive according to questionnaire and parasitology at location 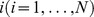. The proportion of children reporting BIU was modelled using a hierarchical logistic regression model (Equation 1) where α is the intercept, 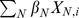 is a vector of *N* predictor variables measured at each location 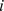 multiplied by their coefficients and 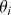 a geostatistical random effect modeled using an isotropic, stationary exponential decay function: 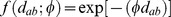 where 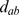 is the straight-line distance between pairs of points *a* and *b*, and 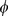 is the rate of decline of spatial correlation. To model the relationship between reported BIU and prevalence of *S. haematobium* infection, we assumed that following an empirical logit transformation, the relationship was linear (Equation 2, Figure 3). This assumption was supported by a likelihood ratio test comparing a linear to quadratic relationship between BIU and *S. haematobium* infection prevalence which showed no difference between models (p = 0.454). The more parsimonious linear relationship was, therefore, assumed. Non-informative priors were used for 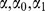 and the coefficients (normal prior with mean 0 and precision 1×10^6^), the prior distribution of 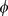 was uniform with upper and lower bounds set at 0.05 and 100 and the precision of 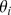 and 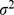 were given non-informative gamma distributions.

Bayesian multivariate logistic regression models, with a non-spatial, unstructured school level random effect, were subsequently generated in a stepwise fashion in WinBUGs version 1.4.1 (MRC Biostatistics Unit, Cambridge and Imperial College London, UK). Covariates that remained significant at the 95% level were included in an identical Bayesian model, replacing the non-spatial random effect for a geostatistical random effect. Covariates that remained significant at the 95% level were then included in the final model.

Model parameter estimates were used to predict the prevalence of *S. haematobium* at schools for which parasitological data were missing. At schools for which only questionnaire data were available, predictions were made using only step 2 of the model, which models the relationship of BIU with infection prevalence. At schools for which only covariate values were known, predictions were made using both steps 1 and 2; step 1 to predict BIU prevalence based on covariate values and step 2 to predict infection prevalence from predicted BIU (Figure 2). Within a Bayesian framework, these predictions are in the form of a posterior distribution, which is formed of the distribution of possible prevalence values a site may take. This feature of Bayesian statistics makes it possible to estimate the probability that the prevalence of *S. haematobium* is greater than any specified threshold.

Following a burn-in of 9,000 iterations, the values for the intercept and coefficients were stored for 1,000 iterations and model convergence was assessed using diagnostic tests for convergence and by visually inspecting the time series plots. Convergence was successfully achieved after 10,000, and the model was run for a further 10,000 iterations with thinning every ten iterations, during which predictions were made.

### Model validation

To evaluate the performance of the final model, each of the three datasets (Figure 1) were first randomly split into 10 subsets. To generate a single set of training data, 9 of the 10 subsets from each of the three datasets were selected. The excluded subsets from each of the three datasets were combined to form the corresponding validation dataset. This process was repeated ten times so that every data point was included once in a validation set. As there were only 6 schools for which only parasitological data were available, these could not be split equally over the 10 validation sets, therefore only a random selection of 6 of the 10 validation datasets contained schools with only parasitology data. For each validation dataset both BIU and infection prevalence was predicted at each location using models built with the corresponding training dataset. To assess discriminatory performance of the predictive model the following validation statistics were calculated: sensitivity; specificity; positive predictive value (PPV); negative predictive value (NPV) and; area under the curve (AUC) of the receiver operator characteristics (ROC; a plot of sensitivity vs. 1-specificity). AUC values of <0.7 indicate poor discriminatory performance, 0.7–0.8 acceptable, 0.8–0.9 excellent and >0.9 outstanding discriminatory performance [20]. For the questionnaire data, the predicted probability of prevalence of blood in urine being greater than 10% and 30% were compared to observed prevalence by questionnaire in the 614 for which questionnaire data were available. For the parasitological data, the predicted probability of prevalence of infection being greater than 10% and 50% were compared to observed prevalence by microscopy in the 33 schools for which parasitology data were available. These thresholds correspond to those used to guide frequency of interventions by questionnaire and microscopy [21]. Where infection prevalence is <10% no action is required, where infection prevalence is ≥10% and <50% treatment should occur biennially and where infection prevalence is ≥50% or reported blood in urine prevalence is ≥30% annual treatment is warranted. Mean error and mean absolute error were used to assess bias and accuracy of predictions respectively.

## Results

**Figure 4:**
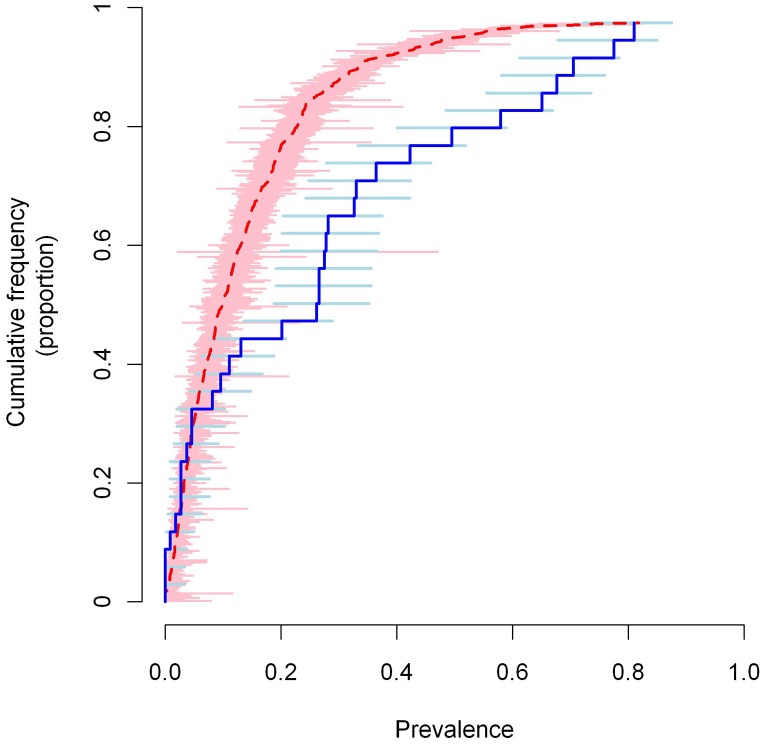
Cumulative frequency plots of the reported blood in urine and infection data. Estimates of the proportion of school children reporting blood in urine in 614 schools is shown in red and estimates of the prevalence of *S. haematobium* infection in 33 schools in blue. Horizontal bars indicate the 95% confidence intervals.

Questionnaire data were available from 312,575 individuals (mean 509.1 per school) and parasitological data were available from 3,486 individuals (mean 105.6 per school). The overall prevalence of reported blood in urine and infection was 12.6% (range 0–81.7%) and 25.6% (0–81%) respectively (Figure 4).

Table 1 presents the results from the Bayesian models including a non-spatial and spatial random-effect. The non-spatial model suggested that three covariates should be retained: EVI, GlobCover and distance to nearest river. Once a spatial random effect was introduced to the model, however, only distance to nearest river remained statistically significant (Table 1). Whilst the non-spatial model produced a lower DIC value than the spatial model (4581.6 vs 4605.6), inspection of the non-spatial random effect at each school revealed significant residual spatial autocorrelation (Moran's I, p<0.001). As residual spatial autocorrelation can overestimate associations between outcomes and covariates due to non-independence [22], a model accounting for spatial autocorrelation between schools was therefore chosen over a non-spatial model. DIC values suggested that a spatial model including distance to water provided a better model fit than one without (4605.6 vs 4610.6) and this was chosen as the final model. This final model suggested that the odds of being infected when being >5 km from the nearest river were nearly half that when <2 km from the nearest river (OR 0.58, BCI 0.30–0.99). The rate of decline in spatial correlation was 22.19 (95% BCI 16.9–28.41) which corresponds to a range (i.e. the distance at which spatial correlation falls to <0.05) of 15 km (95% BCI 11.7–19.7 km).

**Table 1:** Estimates of Bayesian non-spatial and spatial logistic regression models.

Model	Variable	Mean Odds Ratio	95% Bayesian Credible Interval
**Non-spatial**	Enhanced vegetation index (EVI)	0.81	0.74–0.88
	Non-artificial surfaces^1^	1	
	Artificial surfaces	0.42	0.22–0.72
	<2 km from river	1	
	2–5 km from river	0.82	0.69–0.95
	>5 km from river	0.37	0.23–0.56
	Variance of the non-spatial random effect	0.97	0.43–2.12
	DIC	4581.6	
			
	<2 km from river	1	
	2–5 km from river	0.94	0.79–1.1
**Spatial**	>5 km from river	0.62	0.34–0.97
	Range of spatial correlation (km)	15	11.7–19.7
	Variance of spatial random effect	1.45	1.21–1.75
	DIC	4605.6	
			

1 As there appeared to be no difference in risk between non-artificial GlobCover categories (cultivated land, natural and semi-natural terrestrial vegetation, and natural and semi-natural aquatic vegetation) these were combined for analysis.

**Figure 5:**
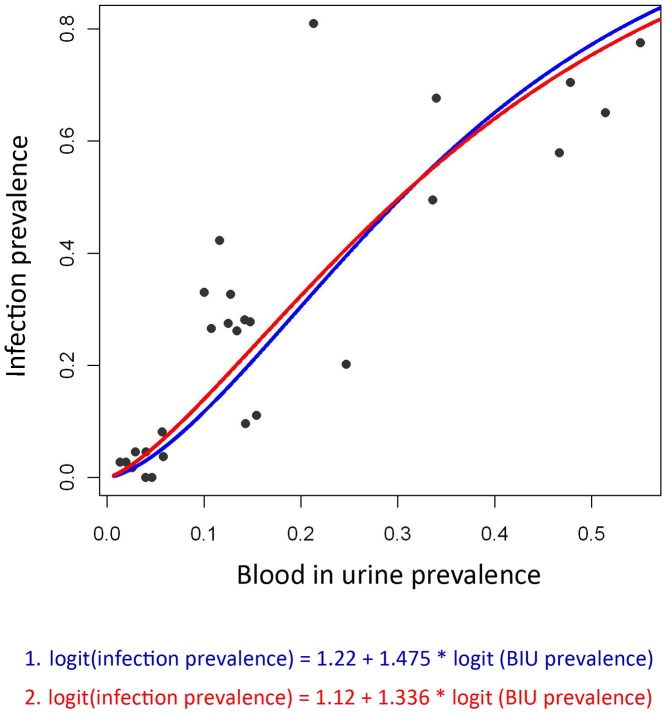
Predicted relationship between the prevalence of reported blood in urine and prevalence of *S. haematobium*. Data are shown for the 27 schools along the Kenyan coast for which both reported blood in urine and infection (by urine filtration) data were available. Blue line indicates the posterior mean of the parameters generated from the non-spatial model parameters (equation 1 – blue) and the red line indicates the posterior mean of the parameters generated from spatial model (equation 2 – red).

The calibration component of the model, which aimed to investigate the relationship between reported blood in urine and parasitology, showed a predictable positive non-linear association between the two indicators (Figure 5). The fact that reported blood in urine consistently underestimated infection prevalence is in line with previous studies and supports the finding that a reported blood in urine prevalence of 30% corresponds to an infection prevalence of 50% [4], the WHO threshold required for annual praziquantel treatment. Interestingly, a reported blood in urine prevalence of 10% appeared to correspond to an infection prevalence of 10%, the WHO threshold for biennial mass treatment [21].

**Figure 6:**
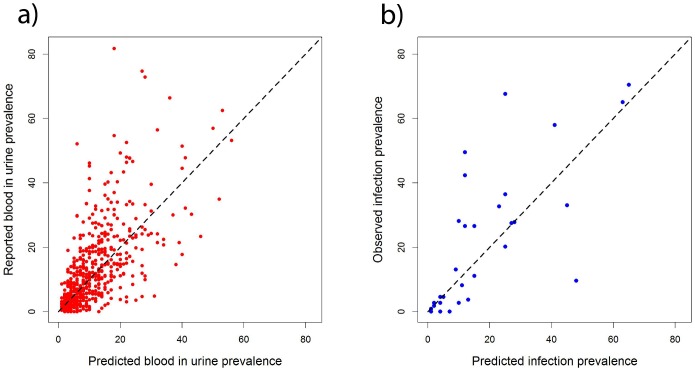
Results of the model validation. Scatter plots of a) reported blood in urine (BIU) prevalence against predicted blood in urine prevalence at all 614 schools for which questionnaire data were available and b) observed infection prevalence against predicted infection prevalence at all 33 schools for which parasitological data were available. Dashed lines refer to a perfect relationship between observed and predicted prevalence.

The model was validated in terms of its ability to predict the prevalence of BIU at the 614 schools with questionnaire data and prevalence of *S. haematobium* infection at the 33 schools with parasitological data (Figure 6). In terms of predicting the prevalence of BIU, the model had reasonable sensitivity and specificity in distinguishing schools according to the 10% treatment threshold, but had very poor sensitivity at the 30% threshold (Table 2). Mean error and mean absolute error indicated very little overall bias and relatively accurate predictions (±6%). When predicting the prevalence of *S. haematobium*, the model had excellent sensitivity and moderate specificity at the 10% threshold, but again poor sensitivity at the higher (50%) threshold. The mean error suggested a tendency to underestimate prevalence and mean absolute error suggested predictions were on average out by ±10%.

**Table 2:** Results of the model validation.

	Threshold		Sensitivity	Specificity	PPV	NPV	AUC
**BIU data**	10%		0.76(0.70, 0.81)	0.81(0.76, 0.85)	0.79(0.73, 0.84)	0.78(073, 0.83)	0.86(0.83, 0.89)
	30%		0.26(0.15, 0.4)	0.98(0.96, 0.99)	0.58(0.37, 0.78)	0.92(0.9, 0.95)	0.85(0.8, 0.9)
	Mean error	0.02					
	Mean absolute error	0.06					

	Threshold		Sensitivity	Specificity	PPV	NPV	AUC
**Infection data**	10%		0.94(0.71, 0.99)	0.69(0.39, 0.91)	0.8(0.56, 0.94)	0.9(0.55, 1)	0.91(0.78,1)
	50%		0.5(0.07, 0.93)	1(0.81, 1)	1(0.09, 1)	0.93(0.76, 0.99)	0.93(0.83, 1)
	Mean error	−0.04					
	Mean absolute error	0.1					

Models were validated using all 614 schools with reported blood in urine data and 33 schools with infection data. PPV – Positive predictive value, NPV – Negative predictive value, AUC – Area under the (ROC) curve. Values in parentheses indicate 95% confidence intervals.

**Figure 7:**
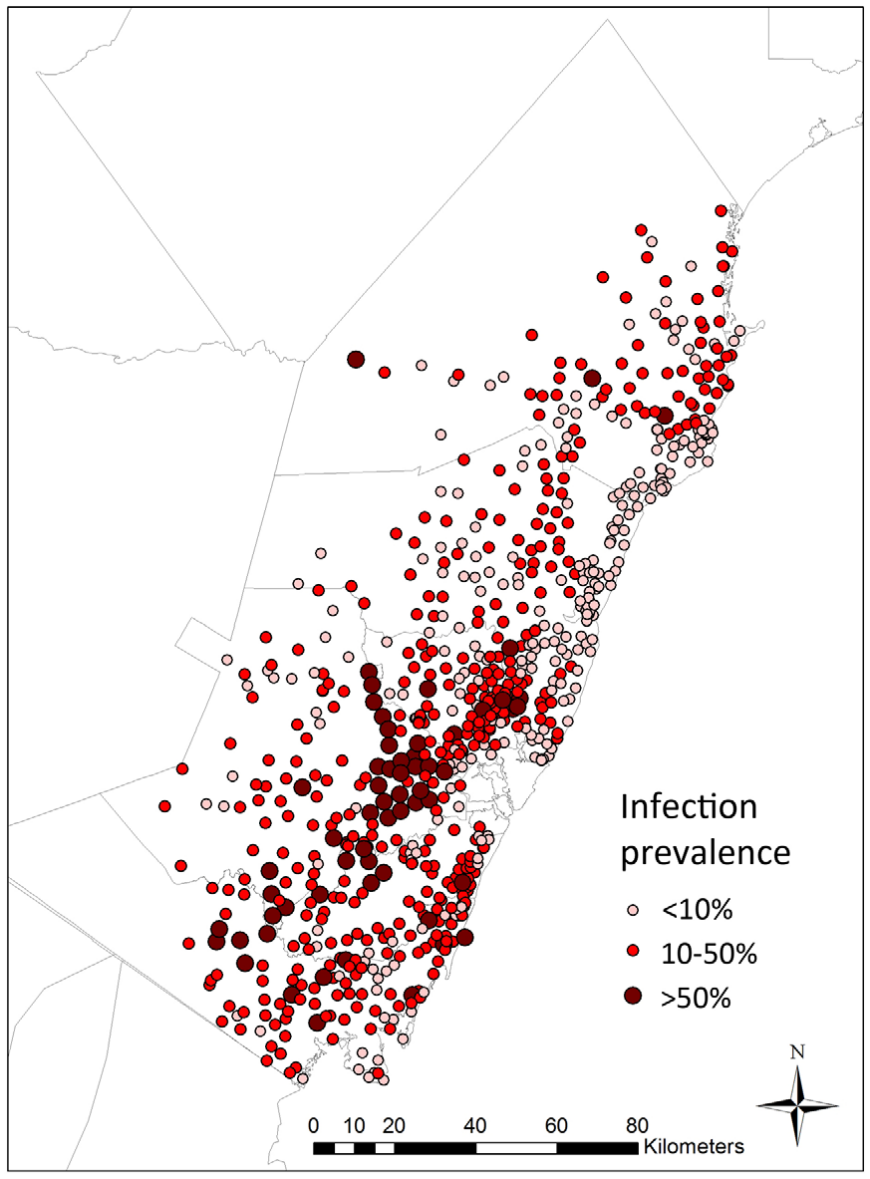
Predicted infection prevalence at schools without parasitological data in Coast province, Kenya. The mean of the predicted posterior of *S. haematobium* infection prevalence is shown.

Figure 7 shows the mean of the predicted posterior of *S. haematobium* infection prevalence at all schools in the study region. Highest prevalence of infection was predicted in the southern inland areas of the coast, and lowest prevalence in the central coastal areas.

## Discussion

Due to the highly focal nature of infection, targeted use of praziquantel is central to the cost-effective control of *S. haematobium*. Whilst questionnaires have been used with success in a variety of settings, imperfect return rates can hinder the targeting of treatment. This study uses bivariate spatial modelling to jointly analyze questionnaire and parasitology data in order to predict the prevalence of *S. haematobium* infection for schools with missing questionnaire data. Whilst the model was unable to reliably discriminate between high and medium risk schools, it was very good at identifying schools that required treatment based on a 10% prevalence threshold.

The success of the model lies in the clear relationship between the prevalence of blood in urine and prevalence of *S. haematobium*, assessed by urine filtration. A new insight provided by the analysis is that a prevalence of BIU of ≥10% is equivalent to a parasitological prevalence of ≥10%. The spatial predictions were also possible due to the observed negative relationship between prevalence of BIU and prevalence of *S. haematobium* and distance to river, a finding consistent with previous studies [12], [23]. The distribution of schistosomiasis is restricted to areas populated by intermediate snail hosts, which are themselves constrained by the availability of freshwater habitats [10]. It is this feature of their biology which results in such a highly focal spatial distribution. The fact that we found no association with other covariates such as land surface temperature, altitude and precipitation, is in contrast to previous studies of *S. haematobium* [7], [9], [23], but is most likely due to the relatively small scale of the study area which results in a relatively homogeneous landscape.

The final model displayed low sensitivity when identifying high risk schools (i.e. those with a reported blood in urine prevalence of ≥30% or infection prevalence of ≥50%). This is most likely due to the relatively low numbers of such schools which, in a statistical sense, become outliers. In the absence of suitable covariates, geostatistical models, which spatially interpolate the school level random effect, will only accurately predict those high prevalence schools if they are in very close proximity to other high prevalence schools. Due to the highly spatially heterogeneous nature of infection, this is quite often not the case and high prevalence schools are either beyond the scale of spatial autocorrelation, in this case up to just 15 km, or are near to schools with lower prevalence, resulting in an underestimation of prevalence.

High levels of performance, combined with their low cost, make questionnaires a highly cost-effective approach as a community level diagnostic tool [3], [24], [25]. Efforts should be made to maximise the return rates of questionnaire, but instances where this is not possible, the current modelling approach enables prediction of prevalence in schools without questionnaire data, on the basis of the calibration of the questionnaire survey and ecological correlates of infection. In addition to this use and the prediction of the prevalence of *Loa loa* [15], bivariate modelling has the potential to improve mapping and decision making for a number of diseases for which rapid diagnostic tests are used alongside other ‘gold standard’ techniques. For example, diagnosis of infection with *Wuchereria bancrofti*, the parasite causing lymphatic filariasis, is often made using an Immunochromatographic Test (ICT) cards which detect circulating filarial antigen [26]. Such tests are cheap, easy to use and display adequate levels of performance making them the field diagnostic of choice [27], [28]. Modelling ICT data together with gold standard infection data from night time bleeds and/or PCR [29], both of which are time consuming, expensive and technically challenging, would allow more accurate predictions of infection prevalence. Such calibration of less than perfect ICTs is likely to become increasingly important as elimination programmes decide where and when to withdraw MDA [28]. Likewise, malaria prevalence estimates are often generated from rapid diagnostic tests (RDTs), microscopy and PCR data which could be modelled together using this approach.

A major limitation of the present analysis is that parasitological infection was based on urine filtration and this approach may have missed some infections, especially those of light intensity. This is particularly true in chronically infected adults within whom the passage of eggs over time causes the development of lesions and fibrous tissue that trap eggs [30]. Newer, more sensitive molecular and immunological methods for the detection of infection are being developed [31], [32] and statistical methods, such as latent class modeling [33], offer a number of advantages and investigation of their use in future analyses is encouraged.

A second limitation relates to the use of the proxy measure distance to river as a risk factor for schistosome infection. In reality, transmission is restricted to areas around specific water bodies inhabited by *Bulinas* spp. snails, and while spatial data relating to the locations of such transmission sites would be ideal, such information is typically available through small-scale research studies and is not available across large spatial scales. Notwithstanding the potential limitations of using distance to river as a risk factor, the results of our modeling suggests that it can increase the statistical robust of the final model. Furthermore, the predictive accuracy of this model is greatly improved by inclusion of a spatially varying random effect which allows the underlying risk surface based on distance to river to be modified at any point according to observed prevalence of BIU in neighbouring schools. Predictions at schools that lie near to transmission sites should, therefore, be adjusted in accordance with higher prevalence of BIU observed at neighbouring schools.

A third limitation relates to the availability of covariate data which varied in spatial resolution between variables (between 300 m for GlobCover data and ∼1 km for WorldClim data). Whilst it is possible to aggregate the higher resolution data to match the lowest resolution data to form a unitized dataset, given the loss of information and the desire to predict over fine scales, this was not undertaken. Similarly, there was a temporal difference between collection of covariate and disease data, with parasitological and questionnaire data collected some years after the covariates. Unfortunately the relevant covariate data were not available for the same period as the epidemiological surveys, but we feel that it is unlikely that these variables would have changed substantially over the short time period of the study, especially as no large-scale intervention had been previously implemented in the study area

In conclusion, here we demonstrate how Bayesian bivariate spatial modelling can model the relationship between the prevalence of report blood in urine and the prevalence of *S. haematobium* infection and, in conjunction with environmental data, predict the small-scale distribution of infection. While the approach was shown to be less reliable in distinguishing between moderate and high prevalence schools, it reliably identified schools requiring mass treatment. In doing so, the approach helps overcome incomplete returns of questionnaires distributed through the existing school system and helps support the rational targeting of schistosomiasis control.

## Supporting Information

Checklist S1 STROBE checklist. Checklist of items that should be included in reports of cross-sectional studies.
(DOCX)
