# Supplementary material for: The Use of Bivariate Spatial Modeling of Questionnaire and Parasitology Data to Predict the Distribution of Schistosoma haematobium in Coastal Kenya
Source: PLoS Negl Trop Dis. 2013 Jan 24;7(1):e2016. doi: 10.1371/journal.pntd.0002016 (PMC3554572; doi:10.1371/journal.pntd.0002016)
Supplement: Checklist S1 — STROBE checklist. Checklist of items that should be included in reports of cross-sectional studies. (DOCX) [file pntd.0002016.s001.docx]

STROBE Statement—Checklist of items that should be included in reports of ***cross-sectional studies***

|  | Item No | Recommendation |
| --- | --- | --- |
| **Title and abstract** | 1 | (*a*) Indicate the study’s design with a commonly used term in the title or the abstract p2 |
|  |  | (*b*) Provide in the abstract an informative and balanced summary of what was done and what was found p2 |
| Introduction | | |
| Background/rationale | 2 | Explain the scientific background and rationale for the investigation being reported p3 |
| Objectives | 3 | State specific objectives, including any prespecified hypotheses p3 |
| Methods | | |
| Study design | 4 | Present key elements of study design early in the paper p3-6 |
| Setting | 5 | Describe the setting, locations, and relevant dates, including periods of recruitment, exposure, follow-up, and data collection p3-4, figure 1 |
| Participants | 6 | (*a*) Give the eligibility criteria, and the sources and methods of selection of participants p3-4 |
| Variables | 7 | Clearly define all outcomes, exposures, predictors, potential confounders, and effect modifiers. Give diagnostic criteria, if applicable p4 |
| Data sources/ measurement | 8* | For each variable of interest, give sources of data and details of methods of assessment (measurement). Describe comparability of assessment methods if there is more than one group p4 |
| Bias | 9 | Describe any efforts to address potential sources of bias p4-5 |
| Study size | 10 | Explain how the study size was arrived at n/a secondary data |
| Quantitative variables | 11 | Explain how quantitative variables were handled in the analyses. If applicable, describe which groupings were chosen and why p4 |
| Statistical methods | 12 | (*a*) Describe all statistical methods, including those used to control for confounding p4-6, figure 2 |
|  |  | (*b*) Describe any methods used to examine subgroups and interactions p4-6 |
|  |  | (*c*) Explain how missing data were addressed n/a |
|  |  | (*d*) If applicable, describe analytical methods taking account of sampling strategy p5 |
|  |  | (*e*) Describe any sensitivity analyses n/a |
| Results | | |
| Participants | 13* | (a) Report numbers of individuals at each stage of study—eg numbers potentially eligible, examined for eligibility, confirmed eligible, included in the study, completing follow-up, and analysed p6 |
|  |  | (b) Give reasons for non-participation at each stage n/a |
|  |  | (c) Consider use of a flow diagram figure 2 |
| Descriptive data | 14* | (a) Give characteristics of study participants (eg demographic, clinical, social) and information on exposures and potential confounders p6 |
|  |  | (b) Indicate number of participants with missing data for each variable of interest n/a |
| Outcome data | 15* | Report numbers of outcome events or summary measures p6 |
| Main results | 16 | (*a*) Give unadjusted estimates and, if applicable, confounder-adjusted estimates and their precision (eg, 95% confidence interval). Make clear which confounders were adjusted for and why they were included p6, table 1 |
|  |  | (*b*) Report category boundaries when continuous variables were categorized p4 |
|  |  | (*c*) If relevant, consider translating estimates of relative risk into absolute risk for a meaningful time period n/a |
| Other analyses | 17 | Report other analyses done—eg analyses of subgroups and interactions, and sensitivity analyses p7, table 2 |
| Discussion | | |
| Key results | 18 | Summarise key results with reference to study objectives p7-8 |
| Limitations | 19 | Discuss limitations of the study, taking into account sources of potential bias or imprecision. Discuss both direction and magnitude of any potential bias p8-9 |
| Interpretation | 20 | Give a cautious overall interpretation of results considering objectives, limitations, multiplicity of analyses, results from similar studies, and other relevant evidence p9 |
| Generalisability | 21 | Discuss the generalisability (external validity) of the study results p8 |
| Other information | | |
| Funding | 22 | Give the source of funding and the role of the funders for the present study and, if applicable, for the original study on which the present article is based Additional Info |

*Give information separately for exposed and unexposed groups.

**Note:** An Explanation and Elaboration article discusses each checklist item and gives methodological background and published examples of transparent reporting. The STROBE checklist is best used in conjunction with this article (freely available on the Web sites of PLoS Medicine at http://www.plosmedicine.org/, Annals of Internal Medicine at http://www.annals.org/, and Epidemiology at http://www.epidem.com/). Information on the STROBE Initiative is available at www.strobe-statement.org.
